# Differential Diagnosis, Clinical Characteristics, and Interventions of Braid-Like Coronary Artery: Case Series Analysis Based on Optical Coherence Tomography

**DOI:** 10.1155/2020/1031675

**Published:** 2020-10-27

**Authors:** Wen-Xiu Leng, Huan-Huan Wang, Hai Ming Liu, Ying Song, Lian-Jun Xu, Jing-Jing Xu, Xue-Yan Zhao, Xiao-Yan Tan, Rong Li, Zhan Gao, Li-Jian Gao, Jue Chen, Jin-Qing Yuan, Yue-Jin Yang, Ji-Lin Chen

**Affiliations:** ^1^Geriatric Cardiology Department of the Second Medical Center and National Clinical Research Center for Geriatric Diseases, Chinese PLA General Hospital, Beijing 100853, China; ^2^Fuwai Hospital, National Center for Cardiovascular Diseases, Chinese Academy of Medical Science and Peking Union Medical College, Beijing 100037, China; ^3^Dehuishi People's Hospital, Changchun, Jilin Province 130300, China; ^4^PLA Rocket Force Characteristic Medical Center, Beijing 100088, China

## Abstract

**Aim:**

Based on optical coherence tomography (OCT), we aimed to determine the diagnosis, clinical characteristics, and interventions of braid-like coronary arteries, which are rare and tend to be diagnosed as a woven coronary artery (WCA) anomaly.

**Methods and Results:**

We identified braid-like lesions on coronary angiography (CAG) in 7 patients (6 men; median age 47 years; age range 26 to 57 years). All patients were heavy smokers. Four patients were diagnosed with an old myocardial infarction and the other 3 with unstable angina. The braid-like lesions were located in the left anterior descending arteries in 2 patients and in the right coronary arteries in the other 5. TIMI grade 2 flow was observed in all involved vessels. OCT findings of all lesions were consistent with recanalization of organized thrombi, which consisted of septa that divided the lumen into multiple small cavities communicating with each other. No separate three-layered structure could be defined. Based on the significance of the stenosis and its related symptoms, drug-eluting stents were implanted in all of the lesions. All patients experienced symptomatic improvement after the intervention and were followed up event-free for 12 months.

**Conclusions:**

Braid-like coronary arteries are likely to undergo recanalization of organized thrombi rather than WCA according to our OCT findings. The majority of cases affect men who smoke heavily. Percutaneous stent implantation may be beneficial in selected patients when feasible.

## 1. Introduction

Braid-like coronary artery is a rare condition that can be seen on coronary angiography occasionally. In such cases, the angiographic imaging is characterized by the branching of a major epicardial coronary artery into thin channels, which then merge again distally into a normal conduit. The twisting course of the multiple thin channels along the vessel causes a braid-like image. Sane et al. first described such a lesion as a woven coronary artery (WCA) in 1988 and defined it as an extremely rare congenital anomaly with a benign manifestation. [1] Since then, a few isolated cases of WCA had been reported. However, the majority of these reports were just based on CAG, and some have been found to be associated with atherosclerosis, chronic ischemia, acute coronary syndrome, and even cardiac arrest [2–12]. The diversity of the manifestations of such lesions has caused great confusion about the diagnosis of WCA as well as the treatment strategy.

Apart from WCA, intracoronary thrombus and spontaneous coronary artery dissection can also mimic braid-like imaging. It is crucial to observe the morphology and nature in vivo in order to make a differential diagnosis in the setting of a braid-like vessel on CAG and then to provide information allowing for an appropriate treatment approach specific to the underlying etiology.

In recent decades, high-resolution imaging modalities have provided new insights into lesions that cannot be fully characterized on angiographic imaging [13]. Optical coherence tomography (OCT) is superior to intravascular ultrasonography for delineating the lumen-intimal interface, and it is better for visualizing intimal tears, false lumens, intramural hematomas, and intraluminal thrombi. Therefore, in addition to clinical baseline information and CAG, we have assessed the morphological characteristics on OCT imaging of a series of braid-like coronary cases and their interventional procedures in order to provide a whole picture of dealing with such lesions.

## 2. Methods

From September 2017 to February 2019, all CAGs conducted at Fuwai Hospital were screened. Braid-like coronary arteries were identified by twisting filling defects in a certain segment, mimicking spiral dissection-like lesions on angiography. Two experienced interventional cardiologists made the final decision about the inclusion of each case. Qualitative and quantitative angiographic measurements were performed using standard techniques with automated edge-detection algorithms (CAAS-5, Pie Medical, Maastricht, The Netherlands) at the catheterization laboratory of Fuwai Hospital. This study was approved by the Institutional Review Board Central Committee at Fuwai Hospital, National Center for Cardiovascular Diseases of China, and written informed consent was obtained from each patient.

Baseline medical history, comorbidities, and risk factors, including a history of myocardial infarction, previous revascularization, hypertension, diabetes mellitus, hyperlipidemia, family history of premature coronary artery disease, and cigarette smoking, were identified according to the data of the index hospitalizations. Smoking over 20 cigarettes a day was defined as heavy smoking. Data on cardiac troponins, electrocardiograms, and echocardiograms were also obtained.

OCT was conducted on each patient immediately after the guidewire was successfully placed distal to the lesion. The OCT images were acquired by a nonocclusive technique with a C7XR system (DragonFly catheter and C7XR, Light Lab Imaging). The artery was cleared of blood by continuous flushing with iodixanol 370 (Visipaque, GE Health Care, Cork, Ireland) at a flow rate of 3.0 ml/s.

The intervention strategy was decided based on the OCT findings according to the discretion of the attending physician. Basically, unstable thrombotic lesions, significant stenosis over 80% with related ischemic symptoms, or evidence on a functional test was an indication for intervention. Dual antiplatelet treatment was prescribed for at least 12 months after stent implantation.

All patients were asked to visit our hospital at 12 months for a routine checkup. Information related to cardiovascular events, including myocardial reinfarction, target vessel revascularization, and rehospitalization for acute coronary syndrome, was acquired. A follow-up coronary angiography was encouraged.

## 3. Results

During the study period of 18 months, coronary angiography was performed for 29,863 patients in Fuwai Hospital. We identified 7 patients with a braid-like lesion, of which 6 were men. The median age was 47 years (range 26 to 57 years). Regarding risk factors, all 7 patients were current heavy smokers. Only 1 patient was concomitant with hypertension, 2 with diabetes, and 3 with hyperlipidemia. No family history of premature coronary artery disease was identified.

Tables 1 and 2 summarize the past history and clinical presentation of the index hospitalization of the 7 patients. A history of anterior ST-segment-elevation myocardial infarction (STEMI) was identified in 4 patients (#1, #2, #3, and #6). Among them, a prior percutaneous coronary intervention was performed in patients #1 and #2 in the culprit left anterior descending artery (LAD), and in these patients, a braid-like and occlusion lesion was observed in the right coronary artery (RCA) at that time. The diagnosis of a previous myocardial infarction was retrospective in patients #3 and #6 based on Q-waves in consecutive precordial leads and the corresponding segment hypokinesis discovered recently. Transient symptoms were recollected by the patients to identify the onset time (3 years ago and 10 months ago for patients #3 and #6, respectively). Except for exertional shortness of breath complained of by patient #1, no specific ischemic symptoms could be identified in these patients (#2, #3, and #6). Patients #4, #5, and #7 complained of exacerbation of chest pain without elevation of cardiac enzymes and were diagnosed with unstable angina. Stents were implanted in the LAD and the left circumflex artery (LCX) of patient #5 approximately 5 years ago, but no information about the RCA was available. No invasive assessment had been conducted in patients #4 and #7 before.

Apart from patient #2 who received thrombolysis within 1 hour after onset, other patients with old STEMI (#1, #3, and #6) presented with precordial Q-waves on electrocardiogram and left ventricular anterior segment hypokinesis on echocardiogram. All patients had a normal left ventricular volume and ejection fraction (Table 2).

**Figure 1:**
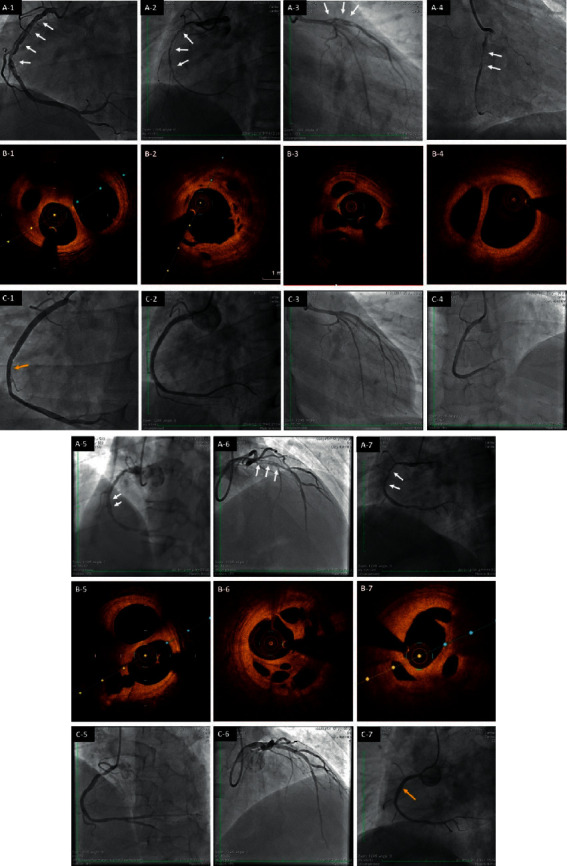
Angiographic and OCT findings of patients #1–#**7**. A1–A7 show the baseline braid-like morphology of the target lesions (white arrow) in patients #1 to #7. B1–B7 show the preprocedural lotus root-like OCT images of the target lesions in patients #1 to #7. C1–C7 show the angiographic results after stent implantation. Side branch compromise after stent deployment occurred in patients #1 and #7 (yellow arrow).

Based on the index CAG, shown in Table 3 and Figure 1, the vessels involving the braid-like segments were the RCA in 5 patients and the LAD in 2 patients. Each lesion appeared to have the arterial lumen divided into multiple thin channels, traversing with a twisting course before merging again into a normal conduit distally. The numerous small tortuous channels formed a braid-like appearance. The average length of the affected segment was 33.3 mm (range 15–60 mm). All of the involved arteries had thrombolysis in myocardial infarction (TIMI) flow grade 2. The previously placed stents in the LAD or LCX in patients #1, #2, and #5 were patent, and stenosis over 90% was found in the RCA proximal to the braid-like lesion in patient #5.

**Figure 2:**
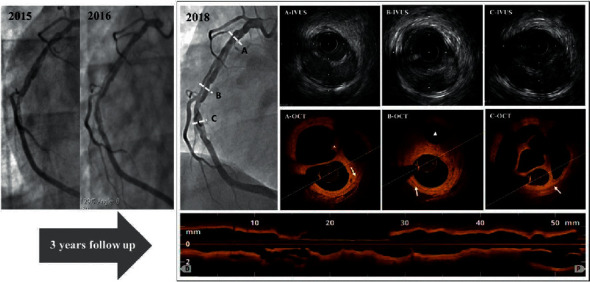
The progress of the braid-like RCA in patient #1 and intracoronary details. Patient #1 experienced acute anterior STEMI in 2015 and a braid-like RCA was discovered incidentally during primary PCI of the LAD. Follow-up CAG in 2016 and 2018 showed little change except for the channel walls becoming smoother and clearer. The cross section intracoronary images drawn from the proximal (A), middle (B), and distal parts (C) are displayed. Intravenous ultrasound (IVUS) detected small channels within the vessel with limited resolution. OCT clearly discerned the signal-rich, high backscattered septa between the microchannels with smooth inner borders and the traces of intraluminal thrombi (star). The three-layer wall structure of the inherent RCA surrounding all of the channels could be defined (arrow). The acute marginal branch originated from a separate channel (triangle).

Notably, this was the third CAG for patient #1. As shown in Figure 2, patient #1 experienced acute anterior STEMI in 2015, and a braid-like RCA was discovered incidentally during primary PCI of the LAD. A follow-up CAG in 2016 showed little change of the lesion. Compared with the former CAGs, the CAG in the index hospital showed little change of the RCA, but the channel walls seemed to be getting smoother and clearer.

After guidewires were successfully placed across the braid-like lesions to the distal part of the vessels, the preprocedural OCT was performed. All of the OCT images turned out to be consistent with recanalization of an organized thrombi, which consisted of signal-rich, high backscattered, and discontinuous septa that divided the lumen into multiple small cavities communicating with each other. [14] These structures had smooth inner borders with traces of intraluminal thrombi. An integrated wall structure of the RCA could be clearly identified surrounding the channels as a whole. However, no separate three-layered structure could be defined inside the walls of the microchannels (Table 3 and Figure 1). There was no definitive evidence of intimal tears or intramural hematomas in the target lesions. The proximal and distal areas adjacent to the lesions were almost normal with mild intimal fibrosis except for patient #5, who had atherosclerotic plagues proximal to the lesion. No plague rupture or dissection was seen.

An intravenous ultrasound test was performed in patient #1 first, and it detected small channels divided by septa within the vessel. However, its resolution was limited. In comparison, the OCT clearly discerned the signal-rich, high backscattered septa between the microchannels and the traces of intraluminal thrombi. Detailed structures of the different segments are shown in Figure 2.

All attending physicians of the 7 patients decided to perform coronary interventions on the involved segments based on the significant stenosis of the true lumen and the culprit or unstable nature of the lesions.  Table 4 lists the details of the interventions. Single- or double-lumen microcatheter systems were deployed in 4 cases, and hydrophilic coated guidewires or those with a polymer cover were used in all 7 cases to successfully cross the lesions. After adequate predilation, drug-eluting stents were able to be deployed in all lesions.

All of the procedural results were perfect except for patients #1 and #7, who had a side branch compromise (Figure 1). Particularly, patient #1 suffered from persistent ventricular fibrillation and lost consciousness during the OCT test. After swift and successful defibrillation, the procedure resumed. The preprocedural OCT confirmed the acute marginal branch originated from a separate lumen different from the true lumen (Figure 2). Based on the predictable high risk of side branch occlusion, a protective wire was placed in the acute marginal branch in advance, and a cutting balloon was used to destroy the separating membrane with high pressure. In addition, the stent was released using the balloon-stent kissing technique. However, the acute marginal branch flow was still compromised to TIMI grade 1 after the jailed wire withdrawal.

None of the patients complained of any chest pain after the procedure. All patients enjoyed symptomatic improvement after the intervention and were discharged as planned (2-3 days after the procedure).

Patients were all followed up event-free at 12 months after discharge. Patients #1 and #3 underwent a follow-up CAG, which showed the stents in the braid-like segments were patent. Of note, TIMI grade 3 flow was restored in the compromised acute marginal branch of patient #1.

## 4. Discussion

Despite their scarcity, braid-like coronary lesions could be encountered by interventional cardiologists in daily practice. As the first case series analysis of braid-like coronary artery, the present study aimed to shed light on their nature, clinical risk factors, and a reasonable treatment approach to such lesions. The results from 7 cases showed braid-like lesions affected predominantly men who were heavy smokers. The lesions mostly involved the RCA followed by the LAD and they compromised blood flow.

OCT imaging provided details of the characteristics of the target lesions, which were consistent with the diagnosis of a recanalized organized thrombi. Based on the significance of the stenosis and related symptoms, drug-eluting stents were implanted in all 7 lesions. All of the patients enjoyed symptom improvement and were event-free at 12 months. Combined with previous case reports, we believe this study has added some important information about lesions presenting with braid-like morphology on CAG.

In previously documented case reports, as shown in Table 5, the most frequent diagnosis for such a braid-like coronary artery was a WCA anomaly [1–10,12,15–17]. However, for most of these cases, we may need a different diagnosis. As is widely acknowledged, WCA is an extremely rare congenital anomaly with an unexplained etiology and benign nature, which was first described thirty years ago on CAG. It was not until 2017 when the first histopathology evidence from a WCA was obtained from a braid-like lesion found incidentally in the RCA of a sudden death patient [11]. This case definitely denied the benign nature of the WCA anomaly, and it seemed crucial to discriminate these lesions by morphology. As the histopathology study disclosed, branches of the WCA anomaly had their own separate 3-layered wall structures that did not communicate with each other.

Obviously, the OCT findings of the present 7 braid-like lesions do not meet the diagnostic criterion of WCA. Looking back at the previously reported cases, we could not find any compelling evidence supporting the diagnosis of WCA in any of them. The diagnosis of WCA made merely by CAG in early reports was certainly arbitrary and not verifiable. Furthermore, the OCT images provided by recent case reports challenged the diagnosis of the “WCA anomaly” since they showed an integrated wall structure of a previously normal RCA instead of separate vessel structures, and instead, thin channels were present [5,6,10]. Such an OCT appearance was also revealed in a braid-like RCA of a case of Kawasaki disease [24].

Clearly, these prior lesions, combined with the present 7 cases, were more likely to be recanalization of organized thrombi, which could also show a honeycomb-like, Swiss cheese-like, or lotus root-like appearance on OCT [13, 14, 18, 22, 25]. Some of these recanalized thrombi also mimicked a braid-like appearance on CAG [14, 18, 20, 22]. In light of the above evidence, we have come to the conclusion that a majority of braid-like coronary arteries could be recanalization of organized thrombi rather than a WCA anomaly.

Our results showed a male predominance in terms of the incidence of braid-like lesions, which is consistent with the previous case reports (male: female, 21:2, see Table 5). The median age was 47 years (range 26–57 years) in our study and 51 years (range 29–78 years) in previously reported cases as a whole. It was hard to ignore that all of our 7 patients were current smokers consuming over 20 cigarettes a day. Furthermore, smoking was the only cardiovascular risk factor for 4 of our patients. It is well established that smoking, as an independent risk factor for coronary thrombotic events, is associated with endothelial dysfunction and increased platelet thrombus formation [26,27]. This may explain the relatively young age of onset (under 26 years in one patient) in this study. Therefore, we supposed smoking is a potential risk factor for braid-like thrombotic lesions.

The braid-like vessels involved the RCA more frequently than the LAD in our study (5:2), and the LCX was not involved. This distribution was in line with previous case reports as a whole (15, 7, and 4 cases in the RCA, LAD, and LCX, resp.). There were also cases with two (2 cases) or three (1 case) coronary arteries simultaneously affected.

Although the functional significance of recanalization of organized thrombi is unclear, previous studies have reported that blood flow through recanalized channels is insufficient [28]. As shown in our study and most other cases, significant luminal stenosis was displayed and blood flow was diminished. The decreased coronary flow reserve of the coronary artery may induce acute ischemia or even cardiac arrest in some circumstances such as sustained tachycardia. What is more, Fujino et al. reported a honeycomb-like structure close to the entry site of a CTO lesion, indicating the formation of an organized thrombus may be a mechanism leading to the formation of a CTO lesion [25]. Recanalization of a cardiogenic embolism could be another potential cause of such lesions [23]. The need for revascularization of unstable braid-like lesions is usually quite clear, whereas their correlation with adverse cardiovascular events and stable lesions needs additional information.

Only a handful of interventional cardiologists have previously tried percutaneous interventions in patients with braid-like lesions [3, 14, 20, 21, 23, 24]. In addition to the undefined indications, the main concern is the failure of crossing the long and tortuous lesion and potential complications. One complication that can be anticipated is side branch compromise as happened in patients #1 and #7 in the present study and one patient reported by Kang et al. previously [14]. When a side branch originates from a channel separate from the one containing the guidewire, the septa would probably be crushed, occluding the ostium after a stent is deployed. Adequate predilation with high pressure by a cutting balloon and other side branch protective techniques may be useful to reduce the incidence of these complications. Anyway, patients #1 and #7 recovered as usual, and the acute marginal branch flow was restored in patient #1, who received a follow-up CAG at 12 months.

On the other hand, crossing such a complicated lesion proved to be a great challenge. Hydrophilic coated guidewires or those with a polymer cover may be preferred in crossing such a lesion. OCT may be useful to guide the procedure. However, as happened in patient #7, frequently filling the vessel with contrast while performing the OCT had a potential risk of inducing ventricular fibrillation, indicating decreased coronary flow reserve in such braid-like vessels. It may be reasonable to fully assess the potential benefits and the risks, as well as available personnel skills, before making a decision about interventions for a braid-like lesion.

The most obvious limitation of the present study was the very limited number of cases involved and the lack of histological confirmation. However, we tried to present some key information about such an uncommon and confusing lesion based on OCT, which is urgently needed. As a single-centered study, the general incidence of braid-like lesions could not be obtained. More data collected in the future would give more information about the pathophysiology and indications for interventions of such lesions. Moreover, we could not provide information about the pathophysiology of the formation of or the progression of such a thrombotic lesion.

## 5. Conclusion

Braid-like coronary artery, though usually described as a woven coronary artery anomaly, is more likely to be recanalization of an organized thrombi according to OCT imaging. The majority of cases affect men who are heavy smokers. The RCA is the most common site. Stent implantation may be beneficial for selected patients, but complications such as side branch compromise should be of concern. More information is needed about the pathophysiology and prognosis of such thrombotic lesions.

## Figures and Tables

**Table 1:** Demographic characteristics, risk factors, and past history.

S/N	Sex	Age (year)	Risk factors					Old MI (time)	Previous PCI		
			HTN	DM	HL	Smoking	FH of PCAD		Timing	CAG findings	Intervention
1	Male	26	o	o	o	Heavy	o	Anterior STEMI (3 y earlier)	4 h after MI	p-LAD occlusion, braid-like p-RCA	LAD stenting
2	Male	47	+	o	+	Heavy	o	Anterior STEMI (2 m earlier)	3 h after thrombolysis	LAD 80% stenosis, p-RCA occlusion	LAD stenting
3	Male	32	o	o	o	Heavy	o	Anterior STEMI (3 y earlier) (deduced from symptoms and ECG)	3 y after MI	Braid-like m-LAD	None
4	Female	56	o	o	o	Heavy	o	—	—	—	—
5	Male	53	o	+	+	Heavy	o	—	5 y earlier	Triple vessel disease	LAD + LCX stenting
6	Male	57	o	o	o	Heavy	o	Anterior STEMI (10 m earlier) (deduced from symptoms and ECG)	—	—	—
7	Male	47	o	+	+	Heavy	o	—	—	—	—

S/N: serial number; HTN: hypertension; DM: diabetes mellitus; HL: hyperlipidemia; FH: family history; PCAD: premature coronary artery disease; MI: myocardial infarction; STEMI: ST-segment-elevation myocardial infarction; PCI: percutaneous coronary intervention; p-: proximal-; m-: middle-; LAD: left descending artery; RCA: right coronary artery; and LCX: proximal-left circumflex.

**Table 2:** Baseline clinical information of the index hospitalization.

S/N	Chief complaint	ECG	Cardiac troponins	Echocardiography			Diagnosis
				LVEDD (mm)	LVEF (%)	Segment hypokinesis	
1	Exertional shortness of breath for 3 months	QS in V2–V6	Normal	57	53	Anterior and apex	Old anterior myocardial infarction; chronic heart failure (NYHA class II)
2	No symptoms; planned revascularization of occluded RCA	Nonspecific T wave inversion	Normal	51	63	None	Old anterior myocardial infarction
3	No symptoms; transferred for revascularization of LAD	Q wave in V2–V5	Normal	48	60	Anterior and apex	Old anterior myocardial infarction
4	Paroxysmal chest pain for 1 month; RCA significant stenosis on CTA	Nonspecific T wave inversion	Normal	45	60	None	Unstable angina
5	Stenting for angina 5 years earlier; exertional chest tightness for 10 days	Nonspecific T wave inversion	Normal	50	52	None	Unstable angina
6	ECG abnormality discovered for 2 months	Q wave in V2–V5	Normal	53	58	Anterior	Old anterior myocardial infarction
7	Exertional chest distress for 2 years, exacerbation for 1 week	Nonspecific T wave inversion	Normal	48	63	None	Unstable angina

S/N: serial number; ECG: electrocardiogram; LVEDD: left ventricular end diastolic diameter; LVEF: left ventricular ejection fraction; AMI: acute myocardial infarction; and CTA: computerized tomography angiography.

**Table 3:** Baseline angiographic and OCT findings.

S/N	Angiographic findings					OCT findings of braid-like lesions				
	Segment involved	Length (mm)	TIMI flow grade	Culprit or not	Other vessels	Morphology	Separate 3-layered structure	Residual thrombi	Intimal tears	Intramural hematoma
1	p-RCA	60	2	o	LAD stent patent	Multiple thin channels divided by thin discontinuous septa	o	+	o	o
2	p-RCA m-RCA	45	2	o	LAD stent patent	Multiple thin channels divided by thin discontinuous septa	o	+	o	o
3	p-LAD	20	2	+	Normal	Multiple thin channels divided by thin discontinuous septa	o	+	o	o
4	m-RCA	20	2	+	Normal	Multiple thin channels divided by thin discontinuous septa	o	+	o	o
5	PLA	15	2	+	LAD and LCX stent patent	Multiple thin channels divided by thin discontinuous septa	o	o	o	o
6	p-LAD	38	2	+	Normal	Multiple thin channels divided by thin discontinuous septa	o	+	o	o
7	p-RCA	35	2	+	m-LCX CTO	Multiple thin channels divided by thin discontinuous septa	o	+	o	o

S/N: serial number; OCT: optical coherence tomography; TIMI: thrombolysis in myocardial infarction; p-: proximal; m-: middle; RCA: right coronary artery; LAD: left anterior descending artery; LCX: left circumflex; PLA: posterior lateral artery; and CTO: chronic total occlusion.

**Table 4:** Information of interventional procedure and follow-up

S/N	Intervention of braid-like lesions					Follow-up at 12 m		
	Key wire	Predilation balloon/pressure_max_	Stenting	Complication	LOS after PCI (day)	Symptoms	Adverse coronary events	CAG
1	SION blue	2.75 × 10 mm cutting/14 atm	+	VT in procedure side branch TIMI 1	3	Exertional SOB improved	None	Stent patent; side branch TIMI3
2	Fielder XT-A	2.50 × 15 mm/14 atm	+	None	2	None	None	—
3	SION blue	2.50 × 15 mm/16 atm	+	None	2	None	None	Stent patent
4	Fielder XT-R	2.50 × 15 mm/14 atm	+	None	2	Paroxysmal chest pain disappeared	None	—
5	SION blue	2.0 × 10 mm/12 atm	+	None	2	Exertional chest tightness improved	None	—
6	SION blue	2.75 × 10 mm cutting/16 atm	+	None	3	None	None	—
7	SION blue	2.50 × 15 mm/16 atm	+	Side branch TIMI 0	3	None	None	—

S/N: serial number; LOS: length of hospital stay; PCI: percutaneous coronary intervention; CAG: coronary angiography; VT: ventricular fibrillation; and TIMI: thrombolysis in myocardial infarction.

**Table 5:** List of previous case reports on braid-like coronary artery.

s/n	Author	Publish years	Sex	Age (year)	Clinical diagnosis	Culprit vessel	Braid-like vessel	Ischemic indication for target lesion	OCT findings		Revascularization of target lesion	Follow-up
									3-layered structure	Residual thrombi		
1	Sane et al. [1]	1988	Female	55	Valvular heart disease	—	RCA	o	—	—	o	—
2	Berman et al. [4]	1990	Male	51	—	—	RCA	Exercise stress+; SPECT+	—	—	o	—
3	Gregorini et al. [7]	1995	Male	60	Unstable angina	—	LAD, LCX	SPECT+	—	—	o	—
4			Male	62	Post-AMI angina	LAD	LCX	—	—	—	o	—
5			Female	45	Post-AMI fibrinolysis angina	LAD	d-LAD	ECG+	—	—	o	—
6	Martuscelli et al. [15]	2000	Male	42	Unstable angina	LAD	RCA	o	—	—	o	LCX-PCI after 4 years with RCA unchanged on CAG
7	Kaya et al. [8]	2006	Male	56	AMI, exertional angina	RCA	RCA	CAG+	—	—	o	—
8	Kursaklioglu et al. [16]	2006	Male	48	Unstable angina	RCA	LCX	o	—	—	o	Event-free for 5 years with LCX unchanged on CAG
9	Yildirim et al. [17]	2000	Male	0.75	Kawasaki disease	—	RCA	o	—	—	o	Event-free for 4 years with RCA unchanged on CAG
10	Cho et al. [18]	2010	Male	50	Anterior-OMI, Stroke	LAD	LAD	o	o	Trace	—	—
11	Kato et al. [19]	2011	Male	60	STEMI	LAD	RCA	o	o	o	o	—
12	Soylu et al. [9]	2012	Male	48	Inferior-OMI	RCA	RCA	Stress SPECT+	—	—	o	Event-free for 2 years
13	Toutouzas et al. [20]	2012	Male	41	Unstable angina	LAD	m-LAD	o	o	o	Stent	—
14	Yuan et al. [12]	2013	Male	62	NSTEMI	LAD	RCA	ECG+	—	—	o	—
15	Bozkurt et al. [6]	2013	Male	52	Anterior-OMI	LAD	p-LAD, D1, m-LAD	o	o	o	o	—
16	Uribarri et al. [10]	2013	Male	78	Stable angina	RCA	RCA	Stress SPECT +	—	o	CABG	—
17	Akyuz et al. [2]	2013	Male	45	Unstable angina	—	LAD + LCX + RCA	Exercise stress+; SPECT−	—	—	o	—
18	Gómez-monterrosas et al. [21]	2014	Male	29	NSTEMI	LAD	LAD	o	o	o	Stent	Event-free at 3 months
19	Sakurai et al. [22]	2014	Male	74	Unstable angina	RCA	m-RCA, d-RCA	Stress UCG + stress SPECT+	o	—	o	—
20	Alsancak et al. [3]	2015	Male	54	Inferior-OMI, stable angina	RCA	RCA	ECG+; UCG + SPECT+	—	—	Stent	—
21	Haraki et al. [23]	2016	Male	59	Anterior-OMI AHF AF	LAD	LAD	SPECT+	—	—	Stent	Event-free and stent patent after 14 months
22	Val-Bernal et al. [11]	2017	Male	39	Sudden death	RCA	RCA	Autopsy	—	—	—	—
23	Nakano et al. [24]	2017	Male	42	Kawasaki disease	RCA	RCA	SPECT	—	—	Stent	—
24	Bi et al. [5]	2018	Male	59	STEMI	RCA	RCA	ECG+; enzymes+	o	o	CABG	—

S/N: serial number; OCT: optical coherence tomography; AMI: acute myocardial infarction; OMI: old myocardial infarction; p-: proximal; m-: middle; d-: distal; RCA: right coronary artery; LAD: left anterior descending artery; LCX: left circumflex; ECG: electrocardiogram; UCG: ultrasound cardiogram; SPECT: single-photon emission computed tomography; AHF: acute heart failure; and AF: atrial fibrillation.

## Data Availability

The clinical and procedural data used to support the findings of this study are included within the article.
